# Tissue-engineered dermal substitutes constructed by human embryonic stem cell-derived fibroblasts facilitate the repair of skin wounds

**DOI:** 10.3389/fbioe.2026.1777175

**Published:** 2026-03-31

**Authors:** Cui Ge, Lu Yin, Liyuan Jia, Shiyue Liu, Mei Sun, Nike Li, Yuan Yu, Fulin Chen, Jihong Cui

**Affiliations:** 1 College of Life Science, Northwest University, Xi’an, China; 2 School of Medicine,Northwest University, Xi’an, China; 3 Key Laboratory of Resource Biology and Biotechnology in Western China Ministry of Education, Xi’an, China; 4 Key Laboratory of Biotechnology of Shaanxi, Xi’an, China

**Keywords:** differentiation, embryonic stem cells, fibroblasts, tissue-engineered dermal substitutes, wound repair

## Abstract

**Background:**

Tissue engineering is an efficient method for constructing functional skin equivalents to treat large-area skin wounds. However, the source of seed cells is limited.

**Objectives:**

Human embryonic stem cell (hESC) derivatives play an important role in tissue engineering. However, whether hESC-derived fibroblasts can be used as seed cells of tissue-engineered dermal substitutes has not been reported.

**Methods:**

In this study, we bypassed traditional induction methods, but rather directly induced *ch*HES-8 cells to differentiate into mesenchymal stem cells (hESC-MSCs) without going through the embryoid body (EB) stage. hESC-MSCs were enriched and treated with connective tissue growth factor (CTGF) to induce their differentiation into fibroblasts (hESC-Fbs). Finally, we seeded hESC-Fbs into collagen gels to construct tissue-engineered dermal substitutes and evaluated their efficacy in treating skin wound *in vivo*.

**Results:**

The hESCs line *ch*HES-8 expressed ALP, NANOG, OCT3/4, and SSEA-3 and formed teratomas containing three germ layers. FACS analysis results showed that hESC-MSCs expressed mesenchymal cell surface markers, including CD29, CD44, and CD105, but not CD34 or CD45, and these cells could be induced to differentiate into adipogenic, osteogenic, and chondrogenic lineages. qRT-PCR and ELISA assays showed that hESC-Fbs expressed high levels of growth factors (including FGF and TGF-β1), extracellular matrix components (such as COL-III, MMP-1, and FN), and the interstitial cell marker vimentin (VIM), similar to human skin fibroblasts (HSF). The mouse skin wounds were successfully repaired by day 20 post-transplantation of tissue-engineered dermal substitutes derived from hESC-Fbs.

**Conclusion:**

hESCs could be directly induced to differentiate into fibroblasts, which could be applied to the construction of tissue-engineered dermal substitutes and skin defect repair. Moreover, this EB-free induction strategy could offer significant advantages for clinical translation, including higher efficiency and better purity.

## Highlights

- hESCs can be directly induced to differentiate into fibroblasts without undergoing the EB stage.
- Fibroblasts derived from hESCs can be applied to the construction of tissue-engineered dermal substitutes.
- The repair of skin defects can be facilitated after transplanting tissue-engineered dermal substitutes constructed by hESC-Fbs.

## Introduction

Skin is most vulnerable to damage due to constant exposure to the external environment. Wounds are primarily classified into acute wounds and chronic wounds (Khwaza and Oyedeji, 2025). Acute wounds typically have a clearly definite time and cause of injury, and undergo a relatively rapid healing process. Examples include incised wounds, abrasions, and thermal burns. Chronic wounds refer to those that, due to various factors, fail to heal following the body’s normal physiological mechanisms (Sen, 2021; Ding et al., 2022). Among them, difficult-to-heal wounds such as large-area thermal burns, severe burns, and diabetic ulcers severely impair patients’ recovery and quality of life, and may even be life-threatening. At present, the most common treatment method for large-area skin wound is still autologous skin grafting (Hu et al., 2015; Kianian et al., 2023). However, this method is limited by factors such as the patient’s physical condition and the finite availability of donor sites. For patients with severe burns or extensive skin wounds, the available donor skin may be insufficient to meet the grafting requirement, often necessitating multiple surgical procedures (Chogan et al., 2023).

Research and development in Tissue Engineered Skin (TES) offers a promising strategy for the repair of skin defects (Wang W. et al., 2025). Structurally, TES is categorized into epidermal substitutes, dermal substitutes, and dermo-epidermal composite skin substitutes. Among these, dermal substitutes demonstrate significant advantages in deep wound repair, long-term functional recovery, and scar control. However, sourcing suitable seed cells remains a bottleneck in TES research and application. Fibroblasts, as crucial seed cells for TES, play an essential role in wound repair through the synthesis of extracellular matrix (ECM) proteins and the secretion of growth factors and cytokines, directly influence the proliferation and differentiation of adjacent epithelial tissues (Shamis et al., 2011). Nevertheless, the source of fibroblasts is insufficient. and currently they mostly come from excised foreskin. The source is not only limited, but the quality of cells from different batches also varies greatly. Moreover, allogeneic fibroblasts are at risk of infectious pathogen transmission, and lose their phenotype and ability to proliferate when cultured *in vitro* (Liu et al., 2017). Therefore, if effective and sufficient fibroblasts can be obtained quickly, artificial dermal substitutes can be better developed based on fibroblast therapy.

Embryonic stem cells (ESCs) are derived from cells in early embryos, specifically at the late blastocyst stage, and possess unlimited self-renewal ability and pluripotency (Gadkari et al., 2014). ESCs have been successfully induced to differentiate into various cell lineages, such as cardiomyocytes, neural cells, osteoblasts, chondrocytes, adipocytes (Liu et al., 2014; Mazzotta et al., 2018; Wang et al., 2019; Byun et al., 2020; Zarei-Kheirabadi et al., 2020). Under specific conditions, hESC-derived basal keratinocytes can construct a pluristratified epidermis and be applied in TES (Guenou et al., 2009). These research results show the great potential of ESCs in the field of tissue engineering. This potential has been primarily explored through two *in vitro* differentiation methods: direct induction and differentiation via an EB stage. Compared to EB-mediated differentiation, direct induction differentiation is more efficient for studies requiring specific, homogeneous cell types. Direct induction bypasses the complex three-dimensional structures that may form during the EB stage, simplifying subsequent steps for isolating and purifying target cells.

Although the emergence of iPSCs has provided a new approach to circumventing the ethical issues associated with hESCs, and theoretically, autologous iPSC-derived transplants could avoid immune rejection, making them suitable for personalized precision medicine (Park et al., 2024). However, iPSCs must be obtained through somatic reprogramming, and the process of differentiation into a specific cell lineage takes a long time (usually weeks to months). In short, using iPSCs requires more complex steps and longer preparation time. In contrast, hESCs exhibit high consistency, making it easier to establish rigorous quality control systems, which facilitates the preparation of large-scale universal products in emergencies or when autologous transplantation is not feasible. Therefore, despite the ethical considerations, under strict regulatory and ethical frameworks, utilizing the robust differentiation potential of hESCs to address the shortage of seed cells remains a more feasible and promising research direction in tissue engineering. However, there are no reports on the use of hESC-derived fibroblasts in tissue-engineered dermal substitutes. The objective of this study is to determine whether hESCs can be directly differentiated into fibroblasts and serve as seed cells for tissue-engineered dermis.

In the current study, we first confirmed the pluripotency of the hESC line *ch*HES-8. Then, we directed *ch*HES-8 to differentiate into mesenchymal stem cells (hESC-MSCs), which were enriched and induced to differentiate into fibroblasts (hESC-Fbs) via CTGF treatment. Finally, we seeded hESC-Fbs into collagen gels to construct tissue-engineered dermal substitutes and evaluated the therapeutic effect of the tissue-engineered dermal substitutes in a nude mouse skin defect repair model.

## Materials and methods

### Cell culture, characterization and animals

The human embryonic stem cell (hESC) line, *ch*HES-8, was kindly provided by Prof. Yan from Northwest University. The cells were maintained on Matrigel (BD Biosciences, San Jose, CA, USA)-coated plates with mTeSR®1 medium (StemCell Technologies, BC, Canada) at 37 °C in a humidified 5% CO_2_ incubator (Thermo Fisher Scientific, Waltham, MA, USA) according to previous protocols (Olee et al., 2014; Zhong et al., 2014). A cell counting kit-8 (CCK-8) (Solarbio, Beijing, China) was applied to analyze cell proliferation. *ch*HES-8 cells were seeded on glass coverslips to assess cellular pluripotency *in vitro*. Cellular immunofluorescence staining of NANOG, OCT3/4 and SSEA-3 was performed as described previously (Liu et al., 2014). Images were taken with a laser confocal microscope (FV3000, Olympus Corporation, Tokyo, Japan).

A total of 25 male nude mice, aged 4 weeks, were provided by the Experimental Animal Center of Xi’an Jiaotong University. All animal procedures were performed in accordance with the guidelines of the Institutional Animal Ethics Committee of Northwest University, and the study was approved by the Ethics Committee (approval No. NWU-AWC-20241005M).

A total of 1 × 10^6^ *ch*HES-8 cells per mouse were injected subcutaneously into the backs of the nude mice to test the pluripotent differentiation capacity *in vivo*. Approximately 8 weeks after injection, all mice were first deeply anesthetized with an intraperitoneal injection of sodium pentobarbital (50 mg/kg) (Feng et al., 2020). Following the confirmation of a complete lack of pedal reflex, cervical dislocation was performed to ensure euthanasia. The formed tumors were harvested, fixed in 4% paraformaldehyde, embedded in paraffin, sectioned at 4 μm, and stained with hematoxylin and eosin (H&E), and Masson’s trichrome (MT) staining. Images were subsequently captured using a light microscope (Nikon, Tokyo, Japan).

### Generation of hESC-MSCs

We directly induced *ch*HES-8 cells to differentiate into mesenchymal stem cells (hESC-MSCs) without going through the EB stage. For differentiating *ch*HES-8 cells toward a mesenchymal lineage, the mTeSR®1 medium was replaced with MesenCult® MSC Culture Medium (Stemcell, Cambridge, U.K.). The cells were cultured for 12 days, and the medium was changed every 3 days. The spindle-shaped cells were selectively collected using a scraper. The acquired cells were centrifuged at 800 rpm for 5 min and cultured on dishes coated with 0.1% gelatin (Gibco, Grand Island, New York, USA). At approximately 80% confluence, the cells were trypsinized and passaged at a ratio of 1:2. The cells were subcultured for additional three passages before use. hMSCs (used as a control) were purchased from ATCC (PCS-500-012^TM^). For all experiments, cells from passage three through passage five were used.

### Flow cytometry analysis of hESC-MSCs

Antibodies (Abs) against CD29, CD34, CD44, CD45, and CD105 and isotype control Abs were all purchased from eBioscience (San Diego, CA, USA). hESC-MSCs at P5 were grown to confluence, harvested and washed with PBS. At least 5 × 10^5^ cells per sample were incubated with an optimal dilution (1:20) of Abs at 37 °C for 60 min in the dark. The cells were analyzed immediately on a flow cytometer (Beckman Coulter, Brea, CA, USA) (Chen et al., 2012).

### Multilineage differentiation of hESC-MSCs

For osteogenic differentiation, hESC-MSCs were treated with osteogenic medium (DMEM supplemented with 10% FBS, 50 μM ascorbic acid, 50 nM dexamethasone, and 10 nM β-glycerophosphate) for 3 weeks. Osteogenic differentiation was tested by Alizarin red S staining and PCR for *OPN* and *RUNX2*.

For chondrogenic differentiation, hESC-MSCs were grown in chondrogenic medium (DMEM supplemented with 10% FBS, 50 μM ascorbic acid, 50 nM dexamethasone, 10 ng/mL TGFβ and 100 ng/mL BMP2). After 3 weeks, the induced cells were processed for Safranin O staining, and PCR assay was used to detect *COMP* and *SOX9* to measure chondrogenic differentiation.

For adipogenic differentiation, hESC-MSCs were cultured in adipogenic medium (DMEM supplemented with 10% FBS, 100 μM indomethacin, 1 μM dexamethasone, 0.5 mM isobutylmethylxanthine, and 10 μg/mL insulin) for 3 weeks. Oil red O staining and PCR for *LEPTIN* and *PPARα* were applied to measure adipogenic differentiation.

All the above reagents were purchased from Sigma-Aldrich (St Louis, MO, USA). The sequences of primers used for PCR analysis are listed in Supplementary Table S1.

### Acquisition of hESC-Fbs

To acquire fibroblast-like cells, DMEM supplemented with 10% FBS, 50 μM ascorbic acid (Sigma-Aldrich, St Louis, MO, USA), and 50 ng/mL recombinant human connective tissue growth factor (CTGF, Prospec, Ness-Ziona, Israel) was used to culture hESC-MSCs instead of MSC culture medium. During the 14-day induction period, the medium was changed every 3 days. Fibroblast-like cells derived from *ch*HES-8 cells were named hESC-Fbs. Human skin fibroblasts (HSF, ATCC PCS-201–012®™) were used as a positive control. The experiment was conducted using hESC-MSCs and HSF cells when the cell confluence reached 70%–80%.

To detect the fibroblasts phenotype of the hESC-Fbs, immunofluorescence staining for VIM and cytokeratin 5 (CK5) (Santa Cruz Biotechnology, Santa Cruz, CA, USA) was performed as described previously (Liu et al., 2017).

Growth factors (including fibroblast growth factor (FGF) and transforming growth factor-β1 (TGF-β1)), extracellular matrix (including collagen III (COL-III) and fibronectin (FN)), matrix metalloproteinase 1 (MMP-1), and vimentin (VIM) levels were measured at 3, 7, 10, and 14 days after induction. Gene expression was determined by quantitative real-time PCR (qPCR) and analyzed using the 2^−ΔΔCt^ method. The PCR sequences of primers are listed in Supplementary Table S2. Protein expression was analyzed by enzyme-linked immunosorbent assay (ELISA). The absorbance value was measured at 450 nm by an enzyme-labeling instrument (Thermo Fisher Scientific, Waltham, MA, USA).

### Preparation of tissue-engineered dermal substitutes

We expanded hESC-Fbs and HSF to construct tissue-engineered dermal substitutes. According to a previously reported protocol (Rao et al., 2016), type I collagen from rat tail was dissolved in 0.1% acetic acid to a final concentration of 4 mg/mL. To prepare tissue-engineered dermal substitutes, 1 mL ice-cold collagen solution mixed with 100 μL cell suspension (5 × 10^6^ cells/ml) was incubated at 37 °C to congeal (Lu et al., 2005). The dermal substitutes were cultured for another 2 days before use. The grafts remain in place over time. For the first 3 days post-surgery, they were covered with Vaseline gauze and adhesive bandages. Animal wounds were monitored throughout the experiment. In addition, cells were labeled with CFSE (Sigma-Aldrich, St Louis, MO, USA) to evaluate cell viability in tissue-engineered dermal substitutes.

### Application of tissue-engineered dermal substitutes in repairing skin defect in a nude mice model

Eighteen male nude mice (six for each group) were intraperitoneally injected with pentobarbital sodium at a dose of 50 mg/kg body weight. A 1.2 cm diameter circular full-thickness defect was created on the back of each mouse, and tissue-engineered dermal substitutes were implanted on the defect immediately. The experiment was divided into three groups: control (collagen gel with no cells), hESC-Fbs (collagen gel with hESC-Fbs), and HSF (collagen gel with HSF) (n = 6 for each group). Images of the wound were taken every 3 days with a digital camera (Canon, Tokyo, Japan) until complete repair. The defect repair percentage was calculated by the following formula: (initial defect area-actual defect area)/initial defect area × 100%. The repaired skin was harvested and fixed in 4% paraformaldehyde, embedded in paraffin, sectioned (5 µm sections). H&E staining and CK5 immunofluorescence staining were performed as described previously (Liu et al., 2017). According to previous reports (Suhaeri et al., 2018), the thickness of the epidermis was measured in the microscopic images of H&E staining, based on three replicates per group and each replicate with three random reepithelialized epidermal regions.

### Statistical analysis

Samples were assayed in triplicate from at least three independent biological replicates. The data are expressed as the mean ± SD of at least three independent samples. The results of RT-PCR were analyzed via Student’s t-test (t-test). One-way Analysis of Variance (ANOVA) was used for other results analysis. *p < 0.05, **p < 0.01, and ***p < 0.001 were considered significant.

## Results

### Characterization of hESCs

**FIGURE 1:**
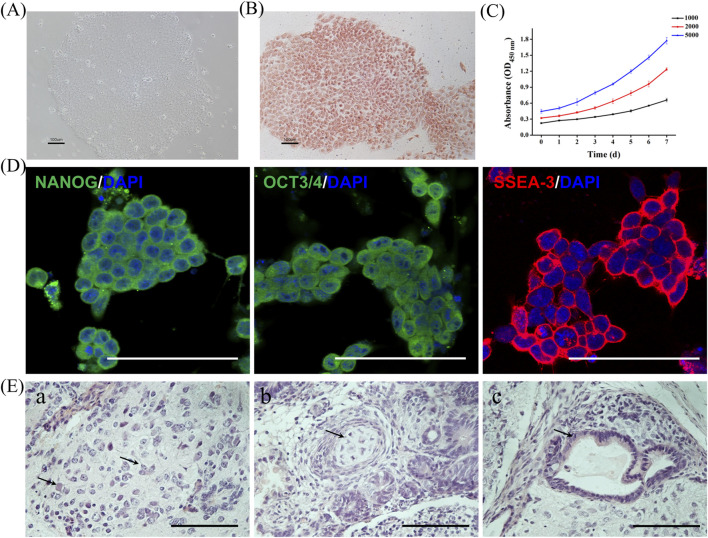
Characterization of Undifferentiated state and pluripotency of *ch*HES-8 cells. **(A)** hESCs cultured in mTeSR®1 medium maintained in an undifferentiated state. Scale bar = 100 μm. **(B)** ALP staining of undifferentiated hESC colonies. Scale bar = 100 μm. **(C)** Growth kinetics of hESCs (data represent mean ± SD, n = 3). **(D)** Immunofluorescence labeling of undifferentiated hESC colonies for NANOG, OCT3/4, and SSEA-3. Scale bar = 100 μm. **(E)** H&E staining of teratoma sections from *ch*HES-8 cells containing nerve cells (**(a)**, ectoderm), cartilage (**(b)**, mesoderm), and gut epithelium (**(c)**, endoderm). Scale bar = 100 μm.

*ch*HES-8 cells displayed clone-like growth (Figure 1A) and expressed high levels of undifferentiated state and pluripotency gene markers, such as ALP, NANOG, OCT3/4, and SSEA-3 (Figures 1B,D). *ch*HES-8 cells exhibited a vigorous proliferative capacity (Figure 1C). Eight weeks after the cells were subcutaneously injected, tumors were observed on the backs of the nude mice. Then, the tumors were collected for histological analysis. H&E staining revealed that the tumor was a teratoma containing three germ layers: the ectoderm (nerve cells), mesoderm (cartilage), and endoderm (gut epithelium) (Figure 1E). All the results demonstrated that the *ch*HES-8 cells used in the present study maintained their undifferentiated state and possessed pluripotency.

### Characterization of hESC-MSCs

**FIGURE 2:**
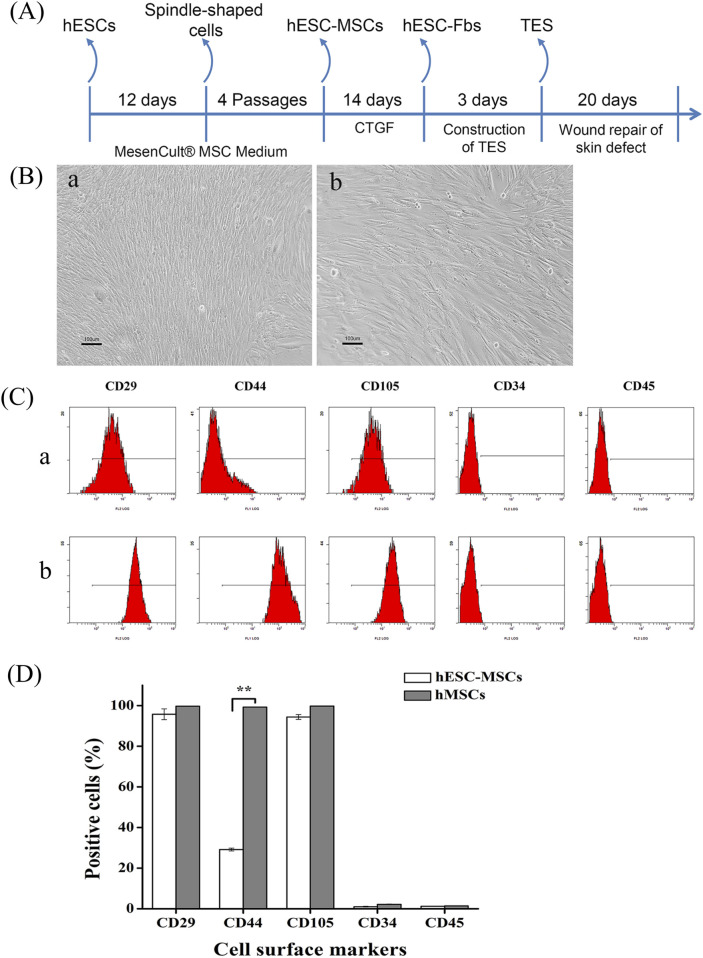
Analysis of MSCs Characteristics. **(A)** Strategy for inducing hESCs differentiation into fibroblasts and constructing tissue-engineered dermal substitutes. **(B)** The spindle-like morphology of hESC-MSCs **(a)** and hMSCs **(b)**. Scale bar = 100 μm. **(C)** Flow cytometric analysis of cell surface antigen expression on hESC-MSCs **(a)** and hMSCs **(b)**. **(D)** Quantitative analysis of MSC cell surface markers (data represent mean ± SD, n = 3). **p < 0.01.

Figure 2A shows the strategy for the induction of hESCs differentiation into fibroblasts to construct tissue-engineered dermal substitutes. hESCs were cultured in MesenCult® MSC medium for 12 days to obtain spindle-shaped cells (Figure 2B). After four passages, the resulting hESC-MSCs were further induced with CTGF for 14 days to become hESC-Fbs. These were then used to construct dermal substitutes and applied to skin defects, with wound repair observed within 20 days. The spindle-shaped cells in Figure 2B were characterized by their cell surface marker expression profiles. FACS analysis revealed that the spindle-shaped cells (hESC-MSCs) expressed mesenchymal cell surface markers, including CD29, CD44, and CD105, but not CD34 or CD45, which was similar to the characteristics of hMSCs. There was a significant difference in the expression of CD44 antigen between hESC-MSCs and hMSCs (Figures 2C,D).

**FIGURE 3:**
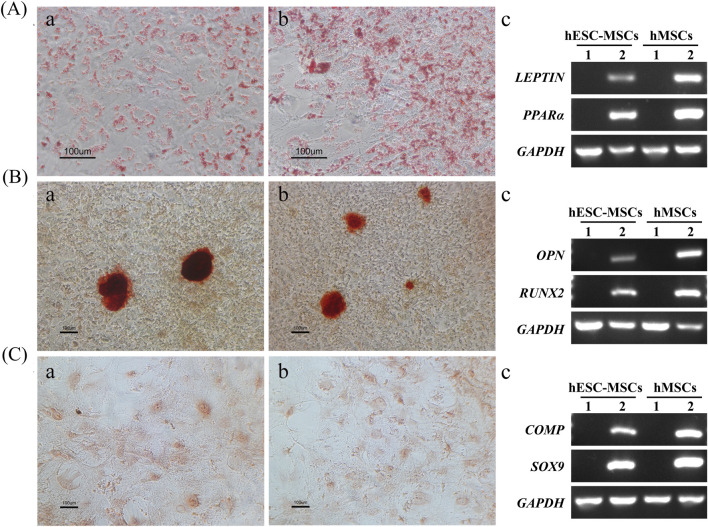
Multilineage differentiation of hESC-MSCs. **(A)** Oil red O staining after adipogenic induction of hESC-MSCs **(a)** and hMSCs **(b)**. PCR analysis of the gene expression profiles related to adipogenic differentiation **(c)**. Scale bar = 100 μm. **(B)** Alizarin Red S staining after osteogenic induction of hESC-MSCs **(a)** and hMSCs **(b)**. PCR analysis of the expression profiles of genes related to osteogenic differentiation **(c)**. Scale bar = 100 μm. **(C)** Safranin O staining after chondrogenic induction of hESC-MSCs **(a)** and hMSCs **(b)**. PCR analysis of the gene expression profiles related to chondrogenic differentiation **(c)**. Scale bar = 100 μm. n = 3.

The osteogenic, chondrogenic, and adipogenic differentiation potential of hESC-MSCs was tested further. After 21 days of adipogenic induction, cells with fat granules in the cytoplasm were observed among hESC-MSCs and hMSCs, which were positive for oil red O staining (Figures 3Aa,b). Additionally, adipogenic relevant genes, including *LEPTIN* and *PPARα* (Qu et al., 2015), were highly expressed, indicating the adipogenic potential of hESC-MSCs (Figure 3Ac). Twenty-one days after osteogenic induction, Alizarin Red staining indicated calcium deposition in the matrix (Figures 3Ba,b). Moreover, the expression of osteogenic markers (*OPN* and *RUNX2)* increased (He et al., 2022), demonstrating that hESC-MSCs have the potential for osteogenic differentiation (Figure 3Bc). After chondrogenic induction of hESC-MSCs for 21 days, Safranin O staining was positive, demonstrating chondrogenic lineage differentiation (Figures 3Ca,b). The expression of *COMP* and *SOX9*, markers of chondrocytes (Dehne et al., 2009), was confirmed to be upregulated by qRT-PCR (Figure 3Cc). The above results demonstrated that hESC-MSCs exhibited the typical differentiation characteristics as hMSCs.

### Fibroblastic differentiation of hESC-MSCs

**FIGURE 4:**
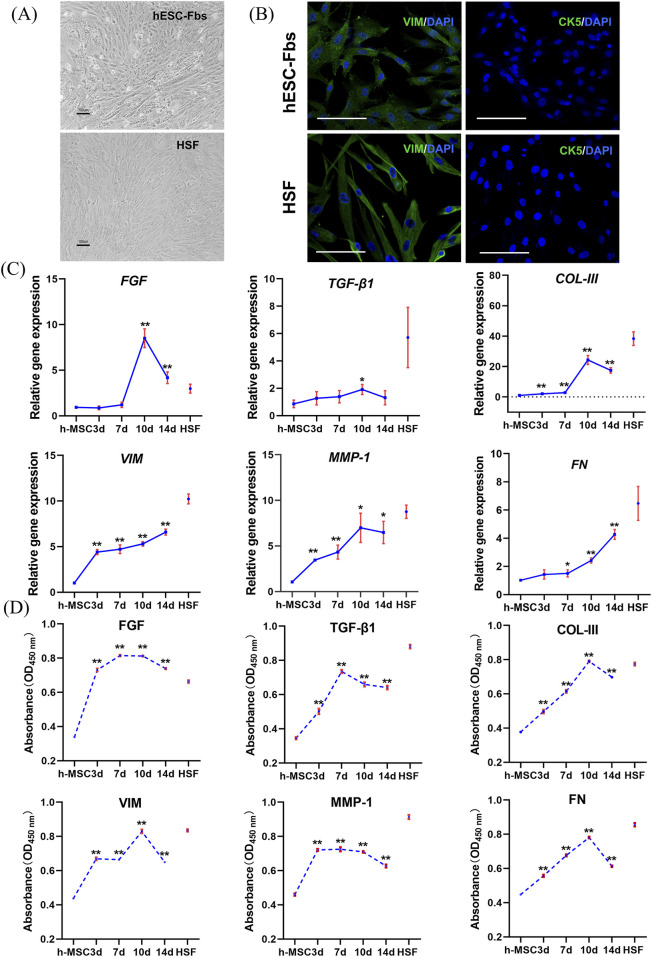
Fibroblastic differentiation of hESC-MSCs. **(A)** Cellular morphology of hESC-Fbs and HSF. Scale bar = 100 μm. **(B)** Immunofluorescence analysis of VIM and CK5 expression in hESC-Fbs and HSF. VIM and CK5 were shown with green, nuclei were counterstained with blue. Scale bar = 100 μm. **(C)** qPCR assay of fibroblast relevant markers during fibroblastic differentiation of hESC-MSCs (data represent mean ± SD, n = 3). *p < 0.05 and **p < 0.01 vs. hESC-MSCs. **(D)** ELISA assay of fibroblast relevant markers during fibroblastic differentiation of hESC-MSCs (data represent mean ± SD, n = 3). **p < 0.01 vs. hESC-MSCs.

In the present study, fibroblastic lineage differentiation of hESC-MSCs was induced by CTGF for 14 days. The morphological appearance of fibroblasts derived from hESC-MSCs was shown in Figure 4A. Fibroblasts synthesize numerous growth factors and extracellular matrix components; therefore, we detected the secretory profiles of hESC-Fbs and HSFs. The expression of several growth factors and fibroblast-related genes and proteins was detected by qPCR and ELISA from day 0–14 after induction. The qPCR results showed that with CTGF treatment, the expression of *FGF, TGF-β1, COL-III,* and *MMP-1* increased at first, peaked at 10 days, and then declined gradually, while *VIM* and *FN* were upregulated continuously (Figure 4C). The ELISA results revealed that the expression trend of most fibroblast-related proteins was basically consistent with that observed by qPCR. The expression of these proteins first increased and then decreased, peaking on day 7 or 10 after induction (Figure 4D). Immunofluorescence staining indicated that hESC-Fbs and HSFs were positive for VIM but negative for CK5 (Figure 4B).

### Tissue-engineered dermal substitutes promoted wound healing in nude mice

**FIGURE 5:**
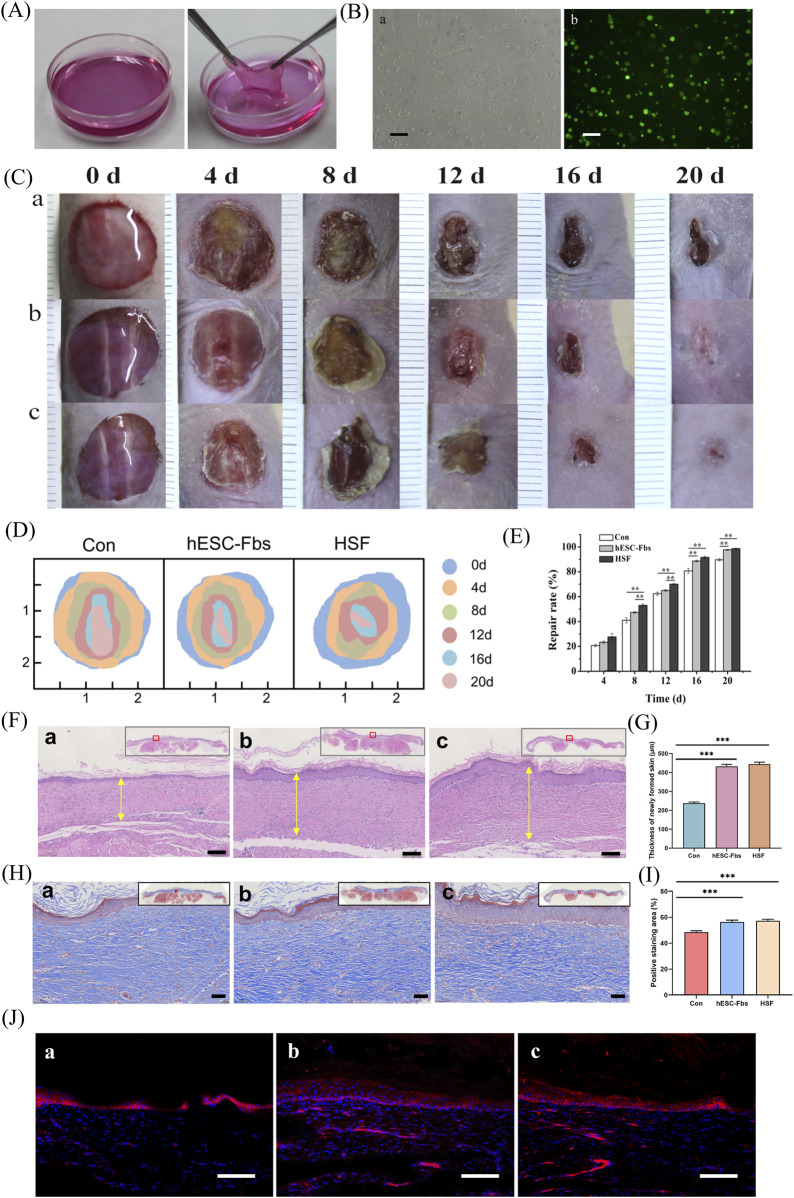
Preparation of tissue-engineered dermal substitutes and its transplantation into nude mice *in vivo*. **(A)** Gross appearance of prepared tissue-engineered dermal substitutes before use. **(B)** Cell morphology in the hydrogels. **(a)** Homogeneous cell encapsulation in the hydrogels. **(b)** CFSE labeling showing the high viability of the cells embedded in the hydrogels. Scale bar = 100 μm. **(C)** Gross appearance of skin defect repair in the control **(a)**, hESC-Fbs **(b)**, and HSF **(c)** groups. **(D)** Schematic diagram of the changes in the injury site over time. **(E)** Wound repair rate of each group in a timely manner. Data represent mean ± SD, n = 6. **p < 0.01. **(F)** H&E staining of repaired skin tissues on day 20 in control **(a)**, hESC-Fbs **(b)**, and HSF **(c)** groups. The yellow arrow indicates the newly formed skin thickness. The gray box in the upper right corner showing the complete tissue slice. Scale bar = 100 μm. **(G)** Statistical analysis of the thickness of the newly formed skin in each group. Data represent mean ± SD, n = 6. ***p < 0.001 vs. Con **(H)** Masson staining of repaired skin tissues on day 20 in control **(a)**, hESC-Fbs **(b)**, and HSF **(c)** groups. The black box in the upper right corner showing the complete tissue slice. Scale bar = 50 μm. **(I)** Bar chart showed quantitative results of the Masson staining (Collagen) analysis. The Student’s t-test was conducted, n = 6. ***p < 0.001 vs. Con. **(J)** Immunofluorescence analysis of CK5 in repaired skin tissues on day 20 from the control **(a)**, hESC-Fbs **(b)**, and HSF **(c)** groups. Red: Ck5; blue: DAPI. Scale bar = 100 μm.

The gross appearance of tissue-engineered dermal substitutes was shown in Figure 5A. The dermal substitutes could be operated mechanically and was so soft that it could be easily made into any shape. Phase-contrast microscopic observation revealed that the cells in the collagen hydrogels were round and homogeneously distributed. Large amounts of green CFSE-labeled viable cells were observed under a fluorescence microscope (Figure 5B), indicating that tissue-engineered dermal substitutes could be generated from hESC-Fbs.

During the process of wound healing, all the animals survived, and no visible inflammation occurred, which was defined as the absence of bacterial infection or purulent discharge at the wound site. In all three groups, the defect areas showed a reduction after tissue-engineered dermal substitutes grafting, and images were recorded digitally on day 0, 4, 8, 12, 16 and 20 (Figure 5C). The wound repair rate after grafting was quantitatively measured, as shown in Figure 5E. The control group exhibited the lowest capacity for wound repair, whereas the hESC-Fbs group and the HSF group exhibited the similar capacity. On day 20 post grafting, the overall wound repair rates of the hESC-Fbs group (97.67%) and HSF group (98.67%) were significantly greater than that of the control group (89.67%) (both *p* < 0.01). Moreover, there was no significant difference between the hESC-Fbs and HSF group. On day 20, the repaired skin tissues were harvested and processed for histological examination (Figures 5F,H). Figure 5G showed that the newly-formed skin’s thickness of the hESC-Fbs group and HSF group was thicker than that of the control group (both *p* < 0.001). The collagen deposition area and intensity were significantly greater in the hESC-Fbs and HSF groups than in the control group (both *p* < 0.001) (Figure 5I). This further indicates that wound healing was faster in these two groups than in the control group. CK5 immunostaining revealed that cytokeratins were present in all the groups, demonstrating that the skin wound had re-epithelialized on day 20 after grafting (Figure 5J).

## Discussion

Although TES offers a promising strategy for skin regeneration, obtaining sufficient quantities and adequate quality of ideal seed cells remains a major bottleneck in its research and application (Bhardwaj et al., 2017; Haxho et al., 2025). Therefore, obtaining a sufficient quantity and quality of seed cells is necessary for the research and application of tissue engineering. The ideal TES seed cells should have strong division and proliferation abilities and low immunogenicity. They can be passed through successive generations without changes in morphology, function, or genetic traits after passage and culture. Currently, most of the seed cells of TES come from foreskin that has been removed. However, the foreskin is a limited source, the quality of cells from different batches varies greatly, obtained cell expansion requires a certain period, and there is also a risk of potential pathogenic infection. Therefore, finding better seed cells is a major demand in the current research of TES.

In the field of skin regenerative medicine, skin organoids and hESC-based TES represent two complementary technological pathways. Organoids are defined as a three-dimensional (3D) cell culture system that is capable of simulating the complexity of real organs with respect to structure and function. This model is primarily derived from adult stem cells (ASCs) or pluripotent stem cells (PSCs) (Huang et al., 2025). Skin organoids have been demonstrated to simulate the natural process of skin development and generate skin accessory structures (e.g., hair follicles), thus indicating their considerable potential in disease modelling and mechanism research (Sun et al., 2021). Nevertheless, Major current issues facing skin organoids include a lack of consistency, challenges with vascularization, and prolonged culture period (Wang Z. et al., 2025). In contrast, TES constructed based on hESC-induced fibroblasts focuses on combining specific differentiated cells with biomaterials to form structurally simplified but functionally clear grafts. It exhibits excellent timeliness and clinical applicability in wound repair, significantly promoting epithelial regeneration and matrix remodeling, but usually cannot reconstruct skin appendages. In the future, with the advancement of biotechnology, skin organoids and tissue-engineered skin analogues will be developed further, providing more powerful tools for research and treatment related to skin regeneration.

In the study of ESCs, directed differentiation *in vitro* has always been a research hotspot. At present, there are two main methods for studying the differentiation of ESC *in vitro*. One method involves the formation of EBs by suspension culture of ESCs, after which different methods are used to induce differentiation into target cells (Sargent et al., 2010). The purpose of the EB pathway is to simulate the process of embryonic development *in vivo*, providing a microenvironment for differentiation and growth, thereby differentiating into various types of cells (Loebel et al., 2003). During EB culture, lack of nutrients or insufficient suspension of EBs will lead to a loose structure and decreased viability, which will seriously affect the further differentiation of ESCs. The three-dimensional structure of EBs makes the differentiated cells contain a large number of cells at different stages of differentiation, which contradicts the principle of high efficiency. Subsequent reports provided another method of ESCs differentiation *in vitro*. ESCs can be directly induced to differentiate (Mazzola and Di Pasquale, 2020). Specifically, ESCs can be cultured in an induction medium or co-cultured with other cell types. The differentiation of ESCs is promoted through the action of cytokines and extracellular matrix components secreted by the co-cultured cells. Cells obtained by direct differentiation were purer. Several types of cells, such as cardiomyocytes (Chen et al., 2023), nerve cells (Chi et al., 2016), liver cells (Ishii et al., 2013), have been reported to be directly differentiated. However, there are no reports on the direct differentiation of ESCs into fibroblasts or their use as seed cells for tissue engineering skin.

In the current study, we tested the method of obtaining fibroblast-like cells from human embryonic stem cells and used them as seed cells to construct tissue-engineered dermal substitutes for the repair of mouse skin wounds. MSC medium was used to culture *ch*HES-8 cells, which were then subcultured for several generations to enrich hESC-MSCs. Notably, compared to bone marrow-derived hMSCs, hESC-MSCs exhibited significantly lower CD44 gene expression (Figures 2C,D). CD44 is a key surface marker of MSCs involved in cell adhesion and migration (Calloni et al., 2013), and its higher expression in hMSCs may indicate greater migratory and adhesive potential. We propose possible explanations for this difference: hESC-MSCs are derived from embryonic stem cells through directed differentiation mimicking early embryonic development, potentially representing a more primitive MSC subpopulation with inherently lower CD44 expression. In contrast, conventionally cultured hMSCs exhibit high CD44 expression after long-term expansion (Qian et al., 2012).

CTGF can regulate cell proliferation, differentiation, adhesion, and migration (Aguiar et al., 2014; Ramazani et al., 2018), it also possesses potent mitogenicity and chemotaxis. Additionally, CTGF can induce fibroblast proliferation and secretion of ECM, and can regulate ECM remodeling (Lipson et al., 2012). MSCs can be induced to differentiate into osteoblasts (He et al., 2022), chondrocytes (Zhou et al., 2019), adipocytes (Qu et al., 2015), muscle cells, nerve cells, and other cell types (Zhao et al., 2012), and can also be induced to differentiate into fibroblasts through CTGF treatment (Yoon et al., 2018). Therefore, we tested whether hESC-MSCs have such differentiation potential. Fibroblasts synthesize numerous growth factors and extracellular matrix components that stimulate and permit cellular proliferation and migration and are critical for skin regeneration (Balaji et al., 2015). After CTGF treatment, we detected an increase in the expression of fibroblast-related factors (including FGF, TGF-β1, COL-III, MMP-1, VIM, and FN) (Figure 4), indicating that hESC-MSCs had differentiated into hESC-Fbs. Notably, the concurrent upregulation of both FGF and TGF-β1 following CTGF stimulation suggests potential cooperative mechanisms in driving fibroblastic differentiation. CTGF is sufficient to induce hMSCs to differentiate into fibroblasts (Lee et al., 2010). FGF-2 acts synergistically with CTGF to enhance fibrogenic differentiation of MSCs (Xu et al., 2017). Additionally, CTGF may exert its effects through activation of specific signaling pathways, including p38 MAPK, which has been implicated in growth factor-induced differentiation of MSCs (Yoshida et al., 2024). Given the expression of related genes and proteins, hESC-Fbs were harvested on day 10 after induction for further animal experiments. Finally, we evaluated the biological activity of tissue-engineered dermal substitutes in a nude mouse skin defect model. *In vivo* experiments showed that tissue-engineered dermal substitutes derived from hESC-Fbs play a role in re-epithelializing skin wound similar to that of normal HSFs. Compared with the cell-free group, the repair ability of hESC-Fbs-derived tissue-engineered dermal substitutes was markedly greater (Figure 5).

## Conclusion

In our study, we found that hESCs could be directed into a fibroblastic lineage via the middle stage of MSCs without going through the EB stage, facilitating defect repair via constructive tissue-engineered dermal substitutes. Compared with the traditional seed cells of TES (fibroblasts extracted from excised foreskin), human embryonic stem cells have advantages in terms of time and technology (easy expansion, easy quality control, etc.). Furthermore, bypassing the EB stage allows for the operationally simpler induction of substantial quantities of fibroblasts from ESCs. In summary, given these characteristics together with the ease and high efficiency of directed differentiation, hESCs are a promising source of cells that could be studied to construct skin substitutes. To promote clinical application, it is currently necessary to conduct systematic preclinical research, such as safety testing and establishing a strict quality control system.

## Data Availability

The original contributions presented in the study are included in the article/Supplementary Material, further inquiries can be directed to the corresponding authors.
