# Supplementary material for: Tissue-engineered dermal substitutes constructed by human embryonic stem cell-derived fibroblasts facilitate the repair of skin wounds
Source: Front Bioeng Biotechnol. 2026 Mar 31;14:1777175. doi: 10.3389/fbioe.2026.1777175 (PMC13076541; doi:10.3389/fbioe.2026.1777175)
Supplement: Supplementary file 1 [file Table1.docx]

***Supplementary Material***

**Table S1. List of primers for PCR of multilineage differentiation relevant genes**

| Gene | Primer sequence: Sense/antisense (5’-3’) | Product size (bp) |
| --- | --- | --- |
| *GAPDH* | GTCAACGGATTTGGTCGTATTG | 212 |
|  | TGGAAGATGGTGATGGGATTT |  |
| *LEPTIN* | TCCTCACCAGTATGCCTTCCAGAA | 180 |
|  | CCACCTCTGTGGAGTAGCCTGAAG |  |
| *PPARα* | GGCTTTACGGAATACCAGTATTTAGGAA | 170 |
|  | CCGCAGATTCTACATTCGATGTTCA |  |
| *OPN* | GGGAAGGACAGTTATGAAACGAGTC | 150 |
|  | GCTGACTTTGGAAAGTTCCTGACTATC |  |
| *RUNX2* | GTAGCAAGGTTCAACGATCTGAGA | 200 |
|  | CGGTCAGAGAACAAACTAGGTTTAGAG |  |
| *COMP* | ACGACTTTGTGGGAGATGCTTGTGA | 174 |
|  | GACTGTCAGGGACTCCGTCATTGTC |  |
| *SOX9* | GCACATCAAGACGGAGCAGCTGAG | 160 |
|  | GGAGTTCTGGTGGTCGGTGTAGTC |  |

**Table S2. List of primers for qPCR of fibroblastic relevant genes**

| Gene | Primer sequence: Sense/antisense (5’-3’) | Product size (bp) |
| --- | --- | --- |
| *GAPDH* | GGTGTGAACCATGAGAAGTATGACAA | 122 |
|  | AGTCCTTCCACGATACCAAAGTTG |  |
| *FGF* | AAGCGGCTGTACTGCAAA | 91 |
|  | TGTGAGGGTCGCTCTTCT |  |
| *TGF-β1* | GAACATCAAGGCAGGCAAAG | 104 |
|  | CTGGGATATGCTGGATGATGAG |  |
| *COL-III* | GCTCTGCTTCATCCCACTATTA | 103 |
|  | CTGGCTTCCAGACATCTCTATC |  |
| *VIM* | GATTCACTCCCTCTGGTTGATAC | 108 |
|  | GTCATCGTGATGCTGAGAAGT |  |
| *MMP-1* | CTCTGACATTCACCAAGGTCTC | 105 |
|  | GATTTCCTCCAGGTCCATCAAA |  |
| *FN* | CTGAGACCACCATCACCATTAG | 107 |
|  | GATGGTTCTCTGGATTGGAGTC |  |
